# Leveraging artificial intelligence to assist the ethical and science-based distribution of COVID-19 vaccines

**DOI:** 10.7189/jogh.11.03124

**Published:** 2021-11-30

**Authors:** Sheng Wu, Andreas Alois Reis, Sameer Pujari, Derrick Muneene, John Reeder

**Affiliations:** 1 Department of Digital Health and Innovation, Division of the Chief Scientist, World Health Organization, Geneva, Switzerland; 2 Department of Research for Health, Division of the Chief Scientist, World Health Organization, Geneva, Switzerland; 3 Special Programme for Research and Training in Tropical Diseases (TDR), Division of the Chief Scientist, World Health Organization, Geneva, Switzerland

While the COVID-19 vaccines are rolling out globally, the battle against COVID-19 has reached a breaking point, where 'decisions made by leaders and citizens will determine when the acute phase of the pandemic will end' [1]. Artificial intelligence (AI) can assist in transforming the conventional decision-making model and assist science-based and ethical distribution of the vaccine.

To ensure timely administration of the vaccine, many countries and local authorities have prepared their COVID-19 vaccine distribution plan, with the prioritized groups and implementation phases identified [2]. The World Health Organization (WHO) has released a value framework and a roadmap for the allocation and prioritization of the COVID-19 vaccine endorsed by SAGE (Strategic Advisory Group of Experts), which are in align with COVAX, the global initiative that ensure rapid and equitable access of COVID-19 vaccine to all countries [3]. However, the distribution of vaccines on ground is facing unprecedented technical complications such as limited supply, unclear duration time, double-injection, special logistic requirements, etc. [3]. While countries and international organizations recognize that central factors in decision-making of vaccine distribution are science-based and ethical [2-4], the conventional static decision-making model is falling short in many ways in meeting this complex scenario. *First,* there are multiple dimensions in the decision-making process, for example, the dimension of objectives and principles of international collaboration mechanism; national and local plans; and the extent of planning activities (WHO listed 10 activities, including planning and coordination, budgeting, regulatory, prioritization, service, training, etc.) [5]. Second, there are uncertain factors in the decision-making process, for example, some key questions about the virus remaining unknown, including the effectiveness and duration of vaccine, and how candidate vaccines under development will progress. Third, the overall epidemiologic setting [4] for the decision-making process is constantly changing and hence is not fully understood. In addition, the deployment of vaccine and its influence on epidemiologic situation is dynamic and needs to be understood in real-time.

**Figure 1:**
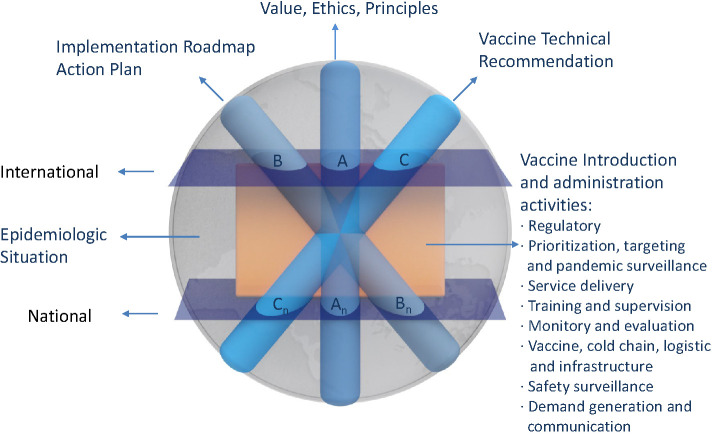
Multiple dimensions of perplexed scientific decision on COVID-19 vaccine distribution scenario in global view. Source: [2-5], author.

**Figure 2:**
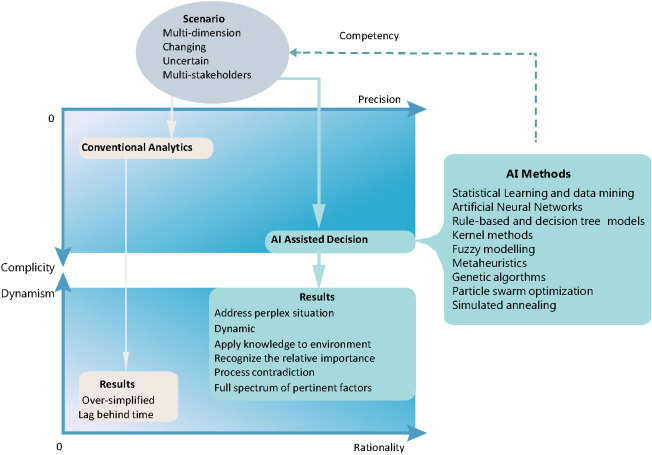
Comparison between conventional analytics and AI assisted decision for COVID-19 vaccine distribution scenario.

Such a complex scenario calls for the most sophisticated methodology and techniques available for decision-making, employing an approach that can accommodate simultaneous consideration of all pertinent factors and aggregate all analytic models involved. Artificial Intelligence (AI), the technology that simulates human intelligence by machines, could transfer the conventional decision-making model by powering Intelligent Decision Support System (IDSS). AI and AI-powered IDSS could assist the science-based and ethical distribution of COVID-19 vaccines, as the essences of AI is a process of optimization under complex, uncertain and dynamic conditions. In general, AI can optimize the operation process by better selecting actions in real-time, coping with uncertainty, reducing stress and information overload, enabling a dynamic response and collaborative decisions [6]. When it comes to assisting distribution of COVID-19 vaccines, it has crucial comparative edge:

One of the key principles WHO and the United Nations have emphasized for fight against COVID-19 is to strengthen solidarity between nations and across all stakeholders, and to recognize that no one is safe, until everyone is safe. Therefore, the decisions on COVID-19 distribution needs to comprehensively address all pertinent multiple dimensions with uncertain and ever-changing factors, in order to achieve effectiveness and robustness (**Figure 1**). For example, in the vaccine-specific dimension, the performance of the existing vaccines remains uncertain, among many other factors, such as delivery logistic capacity, etc. The epidemiologic situation is ever-changing and both global and local view needs to be captured for decision making. The traditional analytical models need already-defined parameters and often simplify a decision process and its scenario, so it cannot reach scientific decision on COVID-19 vaccine distribution. AI methods, such as, Artificial Neural Networks (ANN), fuzzy logic, Case-based Reasoning, evolutionary computing, and Intelligent Agents, etc. can provide powerful support to model a real-time process with multiple-dimension and uncertainty (**Figure 2**).

WHO has issued ethical principles for resource allocation and priority-setting in COVID-19 pandemic, which include 'Equality', 'Utility', 'Prioritize the worst off', and 'Prioritize those tasked with helping others'. It is imperative that the different values be weighed and applied to specific allocation issues using a fair process, which should be transparent, inclusive, accountable and consistent [4]. AI has the ability to accommodate large number of variables and can make sense out of ambiguous or contradictory elements. It can also support large-scale decision making and process large-scale distributed data. These features can contribute to constructing the fair process of ethical decision-making by enabling and improving 'transparency', 'inclusiveness', ‘accountability’ and ‘consistency’.

There has always been disagreement on how to interpret and measure the construct 'ethics' [4,5,]. For example, Emanuel et al. [7] proposed an ethical framework for global vaccine allocation that emphasizes equality ‘requires treating differently situated countries in response to their different needs’ and ‘SEYLL (Standard Expected Years of Life Lost), poverty, and GNI (Gross National Income) should be taken into consideration’, differing from the population-based equality approach adopted by COVAX facility. In Emanuel’s argument, different perspectives referred to were labelled as either ‘mistakenly’ or ‘rightly’. AI techniques can break the conventional dichotomy by accommodating thousands of variables and making sense of them, including contradictory elements, and thus reach maximized. ‘Inclusiveness’ and ‘Consistency’ in decision-making process modelling.

**Figure 3:**
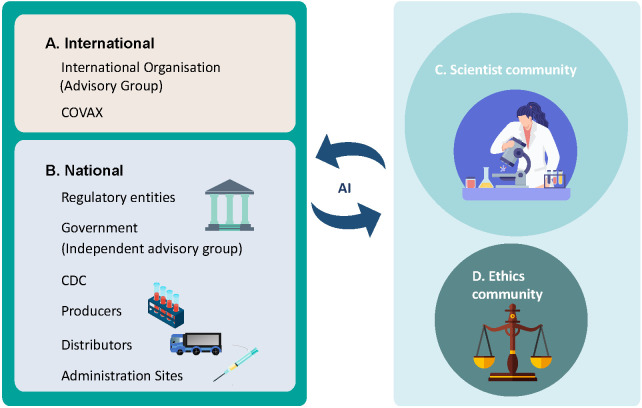
AI assisting scientist community and ethics community integrated large-scale decision making.

Meanwhile, AI can support Large Scale Decision Making (LSDM) and take in all the perspectives of multiple and highly diverse stakeholders and access the provided alternatives with multiple criteria/attributes [8]. Therefore, in addition to conventional stakeholders, the perspectives from the wider scientist community and ethicist community can be genuinely integrated into the decision process by AI, with ‘transparency’ and ‘accountability’ enhanced (**Figure 3**).

**Figure:**
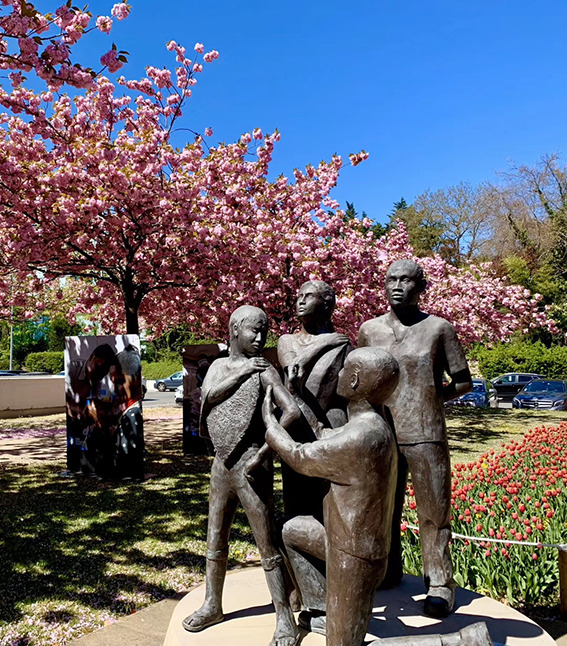
Photo: The statue commentates smallpox eradication in front of WHO office building in Geneva, Switzerland. Prior to eradication, smallpox epidemic was common in many countries, leaving death, blindness and disfigurement behind. The eradication of this devastating, infectious disease has been called one of the greatest achievements in public health. Dr Ren Minghui, ADG, Universal Health Coverage/Communicable and Noncommunicable Diseases, World Health Organization.

The continuous advancement in computing power, algorithms and availability of data has provided launching conditions for leveraging AI for health care to assist in the science-based and ethical distribution of COVID-19 vaccines. Since the COVID-19 pandemic, algorithms have become increasingly common in medical and public health decision-making, with applications ranging from diagnostics to forecasting to resource allocation. In particular, systems based on machine learning increasingly leverage complex forms of data such as images or natural language to perform a range of predictive tasks. [9]. However, a recent trail by Standford University using algorithms for COVID-19 vaccine allocation has provided important lessons learnt: the algorithms resulted in offering vaccines preferentially to senior staff, many of whom were working remotely, compared to only 5 of over 1300 residents who were working in person. which left out frontline doctors, has provided important lessons learnt. [9] The risks of incorrect AI-assisted decision can be mitigated from three aspects ways [10]. First, from the integration of two domains; the risks may occur when the AI developers from computer science field are in lack health domain expertise and knowledge on ground; Second, from the use of data sets, algorithms and models; the risks may occur while defining the “target variable” and “class labels”, labelling the training data, collecting the training data selecting feature and proxies. Third, from “automation bias”; the risk may occur when human decision-makers try to minimise their own responsibility by following the computer’s advice. By close involvement of health domain experts in all phases of AI solution design and development, strengthening legal instruments, including non-discrimination law and data protection law, and by using regulatory instruments, as well as ensuring strict validation mechanism and developing correct understanding the assisting role of AI, these risks can be mitigated.

AI can assist the science-based and ethical distribution of COVID-19 vaccines, as the essences of AI is a process for optimization under dynamic and complicated conditions. According to the Nobel Laureate Herbert Simons′ 'bounded rationality theory', rationality is limited when individuals make decisions. Limitations include the difficulty of the problem requiring a decision, the cognitive capability of the mind, and the time available to make the decision. Humans do not undertake a full cost-benefit analysis to determine the optimal decision, but rather, choose an option that fulfils their adequacy criteria. Applying AI in the decision making of COVID-19 vaccines can push the ‘bounded rationality′ to ‘extreme rationality′, which views the process as a fully rational process of finding an optimal choice given the information available, and it can maximize the impact of COVID-19 vaccine. To realize this revolution in medical and public health decision-making, top in the agenda are strengthening solidarity in data-sharing and cooperation across borders, enhancing collaboration between health domain and the AI community, and accelerating a paradigm-shift in evidence-finding, which will better inform and assist decision-makers.
